# The Management of Brood Mares1Presented to the Minnesota State Veterinary Medical Association, January 10, 1901.

**Published:** 1901-03

**Authors:** J. P. Foster

**Affiliations:** Selby, S. D.


### THE MANAGEMENT OF BROOD MARES.[1]

By J. P. Foster, V.S.,
SELBY, S. D.

This can hardly be called a veterinary subject in every sense of the term, but as many of the large horse-breeding establishments employ veterinarians as superintendents it may not be out of

[1] Presented to the Minnesota State Veterinary Medical Association, January 10, 1901.

place to present a paper on the above subject. On the stock-farm where the only revenue derived from a mare consists in the production of a foal it is necessary for the owner to use his best effort toward getting every one of his mares in foal each year; then, after getting them in foal, the risk of accidents tending to produce abortion should be carefully considered and all the seemingly minor details of every-day management and feeding should be attended to with the utmost care and attention. As regards the ordinary causes of abortion (exclusive of contagious abortion and those that are a sequel to debilitating diseases, such as influenza, pneumonia, etc), I have noticed cases that I am sure were produced by the following causes, as the abortion took place in a few hours after the apparent mishap had occurred:

1. Slipping on icy spots and either falling or producing a strain.
2. Fighting with other horses and getting kicked in the abdomen.
3. Getting pinched in box-stall doors while entering stall (this is where the doors swing out).
4. Getting into deep snowdrifts or muck holes, thereby producing strains.
5. Mares in searching for a place to rub, or, as it seems to be with some of them, from pure curiosity, will get into all kinds of traps, such as between windmill towers, or try to get through some opening that is about half wide enough, and I have seen them get into a feeding pen for sucking colts where they had to get on their knees to crawl under; of course when they come to get out they usually get excited and try to jump over the top, get hung up, and abortion follows.
6. Another cause is abuse from attendants. Some mares are very stubborn and aggravating about going into the barn at night and have to be driven in from the yard by force; then after getting them into the barn it is still harder to run them into their stalls; finally, when they do go into the right stall it is a very natural thing to strike them over the rump, with a halter, board, or anything that comes handy, just as they jump through the door. I once saw this done, causing the mare to fall; this took place while letting them in from the yards at evening and the mare lost her foal that night. Of course there are many other causes that produce abortion, and the foregoing are simply examples of a few of the minor accidents that have come under my notice and might happen at almost any time.

The remedy for this class of accidents is *prevention*. If there is ice in the yard and there is too much of it to be chopped up, ashes or manure may be spread over it and then wet down so that it will adhere and freeze to the ice underneath. Mares that are mean should not be allowed to run with others, and it may be necessary to let them have a small yard by themselves. Box-stall doors should always be fastened open before the mares are let in for the night, and it is wonderful how soon each one will learn her place and seldom make a mistake or get into the wrong stall. In regard to deep snow-drifts in yards, I remember seeing during the severe winter of 1896–97 snow fences in the yards of the largest horse-farm in Iowa. They were built on the plan of snow fences in use on the railways, and were placed around deep drifts to keep the brood mares from getting into the deep snow.

I prefer earth floors in the stalls and alleyways, as the danger of slipping is less than on a plank floor especially in winter, when their feet on very cold days become balled up with snow and ice, sometimes elevating them from the ground three or four inches, and it often seems advisable to knock the balls out of their feet before they are let into the stable. The approaches to the stable door should be arranged so there will be no sill to step over, as a mare will sometimes get just a slight toe-hold with one hind foot in going over a sill and slip off just when the most weight comes on the foot, thereby causing her either to knuckle at the fetlock or causing abnormal extension of the hock and general concussion. As regards diet, we should be careful to see that the food is of the best and that no smutty corn or rusty oats are fed. It is also rather dangerous to allow pregnant mares free access to straw stacks, as is done in the West. Flax straw is particularly harmful, as is any food which acts as a purgative.

It will pay the brood-mare owner to try his mares often in the breeding season, and the plan adopted by most breeding farms of any size is to try all of the mares twice a week, for example, say Wednesday and Saturday, which should be known as "trial days," and on these days each mare is caught and tried. This is kept up until it would be too late in the season to breed them again even if they did come in heat. Most farms keep a "teaser" for this work. This method of trying mares obviates the necessity of figuring out trial days ahead for each individual, as they are all gone over every three days, and if one comes in heat she is sure to be noticed. I have seen mares that were bred in April refuse twice a week from May to July and then come in season. In cases of this kind it is probable that abortion takes place, but owing to the early stage of impregnation the external signs of abortion, such as soiled condition of vulva and tail and tucked-up appearance of the abdomen, are not noticed. Sometimes a mare is noticed in whom it is almost impossible to determine whether she is in heat or not and will allow a horse to tease her and will stand perfectly quiet. The only way to tell is that if she is not in heat she will object if the horse attempts to cover her ; this kind of mare is a source of continual annoyance to all concerned.

In warm weather the best place for a mare to foal is out of doors on a good grassplot, but in the early spring arrangements must be made for foaling inside in a good-sized box-stall, in which there should be plenty of bedding. Mares that foal inside should be watched both night and day, so that in case of non-rupture of the fœtal membranes during labor they can be opened by the attendant before the foal suffocates (this also applies to mares foaling out of doors). Another reason for requiring an attendant is to prevent the mare from lying down with her hind parts against the sides of the stall, thus interfering with the delivery of the foal ; also to catch the foal from those mares that persist in foaling in the standing position, and in so doing preventing the foal from falling and forcibly striking the floor. These are the principal difficulties met with by the attendant, exclusive, of course, of the many different phases of difficult parturition. Immediately after foaling the foal should be carefully placed in one corner of the stall, where the bedding is usually comparatively dry ; the stall should then be thoroughly cleaned out, removing the after-birth and all of the wet straw and drying off the floor beneath as well as possible. The stall should be rebedded with dry straw at once, before the foal attempts to stand, as it is bad policy to allow a young foal to slip and sprawl about on a slippery floor. This should be done regardless of the time of night or how sleepy the attendant may be, and it will be found that small attentions of this kind will go a long way toward making the business a success.

